# Minimal Variation in Functional Connectivity in Relation to Daily Affect

**DOI:** 10.1523/ENEURO.0209-24.2024

**Published:** 2024-12-17

**Authors:** Kate J. Godfrey, Shefali Rai, Kirk Graff, Shelly Yin, Daria Merrikh, Ryann Tansey, Tamara Vanderwal, Ashley D. Harris, Signe Bray

**Affiliations:** ^1^Department of Radiology, University of Calgary, Calgary, Alberta T2N 4N1, Canada; ^2^Department of Psychiatry, University of Calgary, Calgary, AB T2N 1N4, Canada; ^3^Department of Psychiatry, University of British Columbia, Vancouver, British Columbia V6T 1Z4, Canada; ^4^BC Children’s Hospital Research Institute, Vancouver, British Columbia V5Z 4H4, Canada

**Keywords:** affect, dense sampling, functional connectivity, functional magnetic resonance imaging, precision imaging, variability

## Abstract

Reported associations between functional connectivity and affective disorder symptoms are minimally reproducible, which can partially be attributed to difficulty capturing highly variable clinical symptoms in cross-sectional study designs. “Dense sampling” protocols, where participants are sampled across multiple sessions, can overcome this limitation by studying associations between functional connectivity and variable clinical states. Here, we characterized effect sizes for the association between functional connectivity and time-varying positive and negative daily affect in a nonclinical cohort. Data were analyzed from 24 adults who attended four research visits, where participants self-reported daily affect using the PANAS-X questionnaire and completed 39 min of functional magnetic resonance imaging across three passive viewing conditions. We modeled positive and negative daily affect in relation to network-level functional connectivity, with hypotheses regarding within-network connectivity of the default mode, salience/cingulo-opercular, frontoparietal, dorsal attention, and visual networks and between-network connectivity of affective subcortical regions (amygdala and nucleus accumbens) with both default mode and salience/cingulo-opercular networks. Effect sizes for associations between affect and network-level functional connectivity were small and nonsignificant across analyses. We additionally report that functional connectivity variance is largely attributable to individual identity with small relative variance (<3%) accounted for by within-subject daily affect variation. These results support previous reports that functional connectivity is dominated by stable subject-specific connectivity patterns, while additionally suggesting relatively minimal influence of day-to-day affect. Researchers planning studies examining functional connectivity in relation to daily affect, or other varying stable states, should therefore anticipate small effect sizes and carefully consider power in study planning.

## Significance Statement

Brain functional connectivity has been suggested as an affective disorder biomarker, but inconsistent results may be related to affective disorder symptoms varying greatly over short time periods. We used a method called “dense sampling,” where participants are measured at multiple sessions, to characterize how brain functional connectivity and affect vary within people over time. We studied 24 adults who attended four research visits, where they reported feelings of daily affect (mood) and functional connectivity was measured with an MRI scan. We found that functional connectivity is most strongly related to individual differences with minimal influences of daily affect. Future applications of dense sampling to assess variable clinical and nonclinical states must carefully consider study design in anticipation of small effects.

## Introduction

Functional connectivity has been proposed as a potential biomarker for affective disorders (Caseras et al., 2013; Zhao et al., 2023), yet reported associations between clinical symptoms and functional connectivity have limited reproducibility (Taylor et al., 2021). One consideration is that many affective disorders can be described in terms of both trait-like diathesis and substantial state-like symptom variation. Suicidal ideation, for example, can vary widely across short periods of 4–8 h (Kleiman et al., 2017), while also relating to stable risk factors including neuroticism (i.e., a tendency toward negative affect in response to stress; Brezo et al., 2006). Identifying a biomarker of suicidality would therefore require quantifying state-like suicidal ideation fluctuations independent of trait-like neuroticism tendencies. Functional connectivity also has trait-like and state-like qualities, such that whole-brain functional connectivity patterns are primarily driven by group features and trait-like individual variation with smaller contributions from state-like task activations or daily variation (Gratton et al., 2018) . Here, we considered the contribution of daily affective state to varying functional connectivity in an adult community sample (without a clinical diagnosis) as a foundational step toward disentangling stable affective states and traits in clinical populations.

Affect will be defined here as subjective experiences of feeling which include emotions (affective experiences related to overt or implicit engagement with the world) and mood (affective dispositions toward a particular emotional response lasting over hours, days, or weeks; VandenBos, 2015). Networks involved in affective processing have been primarily investigated using task-evoked emotions, which support the importance of default mode, salience, executive control, and visual networks as key functional affective networks. The default mode network (DMN) has been implicated in self-referential thinking (for review, see Raichle, 2015) which may support conceptualization of embodied experiences including evaluation of physiological affective signals (Barrett and Satpute, 2013) or contextualization of affective experiences with prior knowledge (K. A. Lindquist et al., 2012; Roy et al., 2012; Barrett and Satpute, 2013; Barrett, 2017). The salience network is implicated in self-referential processing and monitoring of physiological affective responses (Seeley, 2019). Integration of homeostatic signals by the default mode and salience networks are supported by subcortical regions, particularly the amygdala and nucleus accumbens (Barrett and Satpute, 2013). Executive and control networks may support multiple affect-related processes, including contextualizing affective experiences via episodic recollection (Barrett, 2009, 2006) or orienting attention toward internal evaluation of affective states (K. A. Lindquist et al., 2012). The visual cortex is frequently identified in emotion meta-analyses (Kober et al., 2008; Fusar-Poli et al., 2009; K. A. Lindquist et al., 2012), particularly for negative emotions (K. A. Lindquist et al., 2012). Meta-analysis of task-evoked emotion studies also support the perspective that affect is an interactive process across distributed regions and networks (Barrett, 2017).

One avenue for investigating state-like variability is “precision” functional magnetic resonance imaging (fMRI) and dense sampling, where high volumes of functional imaging data are collected from participants at multiple time points (Arélin et al., 2015; Laumann et al., 2015; Gordon et al., 2017). This approach has been applied previously to investigate associations between time-varying affect and resting-state functional connectivity in a single participant over 73 sessions, each with ∼10 min of fMRI data (Mirchi et al., 2019). Connectivity of default mode network regions, including the precuneus, anterior cingulate cortex, and medial orbitofrontal cortex, were reported to covary with positive affect while connectivity of visual cortex and executive control regions, like the paracentral cortex and lateral prefrontal cortex, covaried with negative affect (Mirchi et al., 2019). Evidence was additionally presented for a multivariate network hypothesis, where greater positive affect generally related to elevated between-network connectivity while greater negative affect generally related to elevated within-network connectivity (Mirchi et al., 2019).

These results provide insight into affect-associated functional connectivity patterns and highlight the potential of dense sampling to examine relations between functional connectivity and time-varying behavioral features, but expansion to larger samples is required to demonstrate the generalizability of findings. Collecting more per-person data additionally increases participant burden, highlighting the value of carefully considering protocol design prior to clinical research implementation. Here, we used dense sampling to examine associations between self-reported positive and negative daily affect and network-level functional connectivity in a community sample of adults. At each of four sessions, participants rated daily affect and fMRI data were collected across three naturalistic passive viewing paradigms with varying levels of engagement. Dynamic naturalistic stimuli were selected here as they can elicit activity in multiple networks, including those involved in emotion, attention, and sensory processing (Bottenhorn et al., 2018), and it has been argued that naturalistic stimuli better replicate real-life cognitive demands while supporting generalizability outside of specific laboratory manipulations (Nastase et al., 2020).

We first considered several univariate hypotheses including relations between affect and within-network connectivity of specific functional networks (default mode, salience, frontoparietal, dorsal attention, visual). We hypothesized that greater positive daily affect would associate with increased default mode network connectivity; greater negative daily affect would associate with increased frontoparietal, dorsal attention, and visual network connectivity; and both positive and negative daily affect would associate with increased salience network functional connectivity. We further considered connectivity between key subcortical affective nodes (amygdala and nucleus accumbens) with default mode and salience networks. We hypothesized that greater positive daily affect would associate with greater subcortex to default mode connectivity and both positive and negative daily affect would associate with greater subcortex to salience network connectivity.

We then considered hypotheses regarding multi-network integration using the multivariate constrained network-based statistic (mv-cNBS) which is a Euclidean distance based approach (Noble et al., 2022). A multivariate effect was examined across the whole brain (all within- and between-network connections), across all within-network connections, and across all between-network connections. In line with previous work (Mirchi et al., 2019), we hypothesized that greater positive daily affect would associate with a general multivariate pattern of elevated between-network connectivity while greater negative daily affect would associate with a general multivariate pattern of elevated within-network connectivity. To support future study design, we additionally characterized the contribution of daily affect relative to other factors which relate to functional connectivity (age, sex, session, viewing condition, head motion, drowsiness).

## Materials and Methods

### Participants

Data reported here were collected as part of a larger precision imaging study which collected repeated imaging and behavioral data from a cohort of children and their parents. In total, 25 parent–child pairs were recruited via community advertisements and from a laboratory participant database. All participants completed four research visits at the Alberta Children's Hospital, approximately 1 week apart, which included completing self-report behavioral surveys and magnetic resonance imaging (MRI). All participants completed a fifth research visit for the purpose of collecting diffusion imaging data which were not analyzed here. Only data from the adult participants were analyzed in the present study. One participant was excluded for excessive motion (criteria outlined below), leaving 24 participants for analysis which are described in Table 1. All parents provided written informed consent for themselves and their child in the first session, and children provided assent. This study was approved by the University of Calgary Conjoint Health Research Ethics Board.

**Table 1.** Participant characteristics

Characteristic	Sample description
Male/female (*n*)	12/12
Age [*M* ± *SD* (range)]	41.15 ± 3.60 (33.75–47.13)
Ethnicity (*n*)	
White/Caucasian	22
Asian	2
Highest education level (*n*)	
High school	6
Bachelor's degree	11
Master's degree	4
Professional school degree	3
PANAS-X positive affect [*M* ± *SD* (range)]	26.51 ± 6.48 (12–39)
PANAS-X negative affect [*M* ± *SD* (range)]	11.86 ± 3.67 (9–30)
Mean framewise displacement [mm; *M* ± *SD* (range)]	0.08 ± 0.03 (0.04–0.20)
Drowsiness [*M* ± *SD* (range)]	2.07 ± 2.11 (0–6)

*n*, sample size; *M*, mean; *SD*, standard deviation; PANAS-X, Positive and Negative Affect Schedule Expanded Form.

### MRI data collection

At each study visit, participants completed a MRI on a 3 T GE 750W scanner at the Alberta Children's Hospital, which included a whole-brain T1-weighted BRAVO (TR, 7.29 ms; TE, 2.66 ms; voxels, 1 mm isotropic) and a series of six runs of T2*-weighted multi-echo, gradient-echo, echo-planar imaging [TR, 2,000 ms; TEs (echo times), 13, 32, 52 ms; number of volumes, 205 (excluding five dummy volumes); flip angle, 70°; in-plane field of view, 220 × 220 mm; in-plane matrix size, 64 × 64; number of slices, 45; slice thickness, 3 mm; slice gap, 0.4 mm; voxel size, 3.4 mm^3^; multiband acceleration factor, 3]. Approximately ∼39 min of fMRI data were collected per session, across six runs. Each run used one of three passive viewing conditions, with each condition repeated, for ∼13 min of fMRI per condition per session. Passive viewing conditions were as follows: (1) a “low-demand video” condition, inspired by Inscapes (Vanderwal et al., 2015), consisting of gentle music and slow-moving footage of naturalistic scenery (e.g., a walk through a canyon, earth from space, and scenes from cities); (2) a “narrative movie” condition consisting of sequential clips from a narrative movie, *Dora and the Lost City of Gold* (2019); and (3) a “non-narrative clips” condition consisting of disparate, short (24–65 s long; mean, 46.4 s) and popular (i.e., viewed > 1 million times) videos accessed on social media platforms (YouTube, TikTok). The order of viewing conditions was pseudorandomized between sessions, and this session-to-session pattern was further pseudorandomized between participants.

### Behavioral data collection

At each visit, participants self-reported daily affective state (over the past 24 h) using the Positive and Negative Affect Schedule Expanded Form (PANAS-X) which includes separate positive affect and negative affect subscales (Watson and Clark, 1994). After each MRI session, participants also self-reported whether they felt tired, sleepy, or had difficulty staying awake during each functional run (i.e., a total of six ratings per imaging session) which were derived from the Amsterdam Resting-State Questionnaire 2.0 (Diaz et al., 2013, 2014) and PROMIS Sleep-Related Impairment Short Form (Yu et al., 2012). A summary score for drowsiness was calculated such that reporting difficulty staying awake was given a score of 3, feeling sleepy was given a score of 2, feeling tired was given a score of 1, and no response was given a score of 0. A score was calculated for each viewing condition by summing across the two runs. Reports of difficulty staying awake were additionally treated as a binary variable to identify runs where participants may have fallen asleep and follow-up analyses were conducted with these data excluded.

### Preprocessing

One low-motion anatomical image for each participant was used for functional registration and surface projection. Structural images were first preprocessed using the recon all pipeline from FreeSurfer version 6 (http://surfer.nmr.mgh.harvard.edu/), after which they were converted to cifti format using the cifti recon all function from Connectome Workbench version 1.5.0 (Glasser et al., 2013). AFNI version 22.1.14 (Cox, 1996) was used on anatomical images, prior to surface projection, to generate white matter and cerebrospinal fluid tissue masks at 10% erosion. Tissue masks were used in functional preprocessing to estimate nuisance signals for denoising as described below.

Functional preprocessing was conducted using in-house Python scripts which used nipype version 1.8.5 (Gorgolewski et al., 2011) to call functions from FSL version 6.0.5.1 (Jenkinson et al., 2012), ANTS version 2.4.0 (Avants et al., 2011), AFNI version 22.1.14 (Cox, 1996), and Tedana version 0.0.12 (The Tedana Community et al., 2022). Preprocessing was first conducted on each echo independently, including FSL *slicetimer* for slicetime correction and FSL *MCFLIRT* for rigid body realignment. Head motion parameters and framewise displacement were estimated on the middle echo time series during rigid body realignment with FSL *MCFLIRT* (Jenkinson et al., 2002)*.* FSL *FLIRT* was used to coregister all echoes within a session to the reference echo (specified as the first echo from the first run of the narrative movie condition), and data for each echo were concatenated within sessions. FSL *FLIRT* was then used for coregistration of data between sessions. Tedana 0.0.12 was then used for optimal combination and to conduct multi-echo independent component analysis for noise mitigation. Tedana automatically classified components as signal, noise, or unknown, and classifications were manually verified and corrected as needed (mean corrections per run, 4.14; range, 0–13). Each optimally combined run was further denoised via linear detrending; bandpass filtering (0.01–0.08 Hz); nuisance regression with head motion parameters and the global, white matter, and cerebrospinal fluid signals as confounds; and censoring (FD threshold, 0.2 mm). Head motion parameters were filtered prior to regression and nuisance signals were estimated by applying anatomical masks to the detrended and filtered data to avoid reintroducing artifacts (M. A. Lindquist et al., 2019). As relatively large consensus regions of interest (ROIs) were used (see details below), spatial smoothing was not performed. Preprocessed functional runs were then warped to the participants’ T1 using ANTS *ApplyTransforms*, and the two runs for each condition were concatenated. Connectome Workbench *cifiti_subject_fmri* function was used to project the preprocessed functional runs to surface space. Data from one subject with excessive motion were excluded at this stage, as 75% of total observations from this subject had mean framewise displacement which exceeded the motion threshold.

### Connectome generation

The Dworetsky Midnight Scan Club Probabilistic Atlas (https://midbatlas.io/; Dworetsky et al., 2021; Hermosillo et al., 2024) from the Masonic Institute of Developing Brain was applied to the preprocessed surface data using the minimum probability threshold of 0.69. This probabilistic atlas was defined using data from the Midnight Scan Club (Gordon et al., 2017) and represents a high-consensus assignment of vertices to networks while excluding vertices with low consensus in network assignment. The minimum probability threshold was selected to include as many vertices as possible during analysis. The data were assigned to 27 high-consensus ROIs from nine functional networks which were the default mode (6 ROIs), salience (1 ROI), cingulo-opercular (5 ROIs), frontoparietal (5 ROIs), dorsal attention (2 ROIs), parietal occipital (2 ROIs), visual (2 ROIs), auditory (2 ROIs), and sensorimotor (2 ROIs). As the atlas includes only one salience network parcel, this parcel was grouped with parcels composing the cingulo-opercular network, which is also implicated in internal self-referential processing and state monitoring (Gratton et al., 2022). The probabilistic atlas included two additional networks, medial temporal lobe (2 ROIs) and temporal pole (2 ROIs), which were not analyzed here due to high dropout (i.e., all observations had varying degrees of dropout in these ROIs). The Tian 3T subcortical atlas (version S4; https://github.com/yetianmed/subcortex; Tian et al., 2020) was additionally applied to assign preprocessed volume data to subcortical parcels from the amygdala (2 ROIs) and nucleus accumbens (2 ROIs). These four ROIs were averaged to estimate subcortical affective regional connectivity. Functional connectivity was calculated as Pearson’s correlation coefficient between parcellated timeseries, which were then Fisher's *Z* transformed.

### Statistical analyses

All statistical analyses were conducted using R version 4.2.1 (R Core Team, 2022). Linear mixed-effects models were conducted using the *lmer* function from the lme4 package version 1.1-31 (Bates et al., 2015).

### Behavior

Summary descriptive statistics (mean and standard deviation) were calculated for continuous variables (affect, age, mean framewise displacement, drowsiness). An association between positive and negative daily affect was investigated using a linear mixed-effects model with random subject intercepts [lmer notation: positive daily affect ∼ negative daily affect + (1|subject)]. Affect was subject mean centered across analyses to allow for estimation of how within-subject variance in daily affect (rather than mixed within- and between-subject variance) is associated with other predictors and with functional connectivity (Raudenbush and Bryk, 2002). Associations between grand mean centered covariates (age, sex, session, mean framewise displacement, drowsiness) and both subject mean centered daily affect measures were also examined in separate mixed-effects models which included random subject intercepts (Brauer and Curtin, 2018). Covariates with multiple scores per session (mean framewise displacement and drowsiness) were averaged within a session for behavioral association analyses.

### Univariate network effects

Linear mixed-effects models were run at each edge to quantify the extent to which functional connectivity associated with positive and negative daily affect (subject mean–centered) which were considered in separate models. Mixed-effects models controlled for the fixed effects of sex, age (grand mean centered), session, viewing condition, head motion (mean framewise displacement, grand mean centered), and drowsiness (grand mean centered) and random subject intercepts [lmer notation for full model: functional connectivity ∼ affect + age + sex + session + viewing condition + head motion + drowsiness + (1|subject)].

For the purpose of statistical inference, network-level observed effects and significance estimates were calculated using the constrained network-based statistic (cNBS; Noble and Scheinost, 2020; Noble et al., 2022). The cNBS statistic was selected here due to reports of increased sensitivity for network-level inference (Noble et al., 2022) and because permutation-based significance estimations avoid the normality assumptions required when comparing to a theoretical probability distribution (Fisher, 1935; Pitman, 1938). To this end, edgewise null distributions were estimated via permutation by repeating mixed-effects models with shuffled outcomes. Specifically, for each edge, functional connectivity was shuffled across sessions, preserving session and subject structure (i.e., all functional connectivity data collected at the same session remained together, and shuffling did not occur between subjects). Network-level observed effect sizes were estimated as the mean across edges within a network, and, at each permutation, a network-level null value was calculated as the mean of permuted edge-level effect sizes. Network-level significance was determined by comparison of the network-level observed effect to the simulated 1,000 permutation network-level null distribution.

Based on the hypotheses described above, network-level effects were investigated for the default mode, salience/cingulo-opercular, frontoparietal, dorsal attention, and visual networks. As hypotheses were directional, one-tailed *p*-values were calculated. In an exploratory analysis, cNBS statistics were additionally calculated for all within- and between-network connections. Any significant findings for the hypothesized and exploratory analyses, considered separately, would be corrected using false discovery rate (FDR) correction. Across analyses, FDR-corrected significance values were only reported in the case of effects that were *p* ≤ 0.05 uncorrected. An estimate of effect size for permutation tests, the standardized effect size (SES), was calculated as the difference of the observed effect from the mean of the permuted null divided by the null standard deviation (Gotelli and McCabe, 2002).

### Multivariate network effects

Multivariate effects across network groupings were investigated using multivariate cNBS (mv-cNBS; Noble et al., 2022). The mv-cNBS statistic estimates a pooled effect across multiple networks by calculating the average Euclidean distance for network-level effect sizes. Significance values for the mv-cNBS statistic were determined by comparison to a mv-cNBS null distribution which was generated by calculating Euclidean distance metrics at each permutation. A whole-brain effect was investigated by calculating a mv-cNBS statistic across all within- and between-network effects. Additional mv-cNBS statistics were calculated for all within-network connections and all between-network connections, based on Mirchi et al. (2019).

### Proportional variance explained

To compare effect sizes for positive and negative daily affect in relation to other factors known to impact functional connectivity, we characterized the degree of variance explained [calculated as conditional *r*-squared (*r^2^c*)]. Variables were introduced to the model in a stepwise fashion, and the difference in *r^2^c* following each step was interpreted as additional variance explained. This process occurred sequentially in separate models for positive and negative daily affect with new predictors being added in the following order: (1) random subject intercepts, (2) demographics (age and sex), (3) viewing condition, (4) head motion, (5) drowsiness, and (6) affect. To represent the data proportionally, the added variance explained was transformed into a percent of the total variance explained. In instances where the addition of a predictor variable negatively impacted model *r^2^c*, the added variance explained was interpreted as zero.

## Results

### Behavior

**Figure 1.**
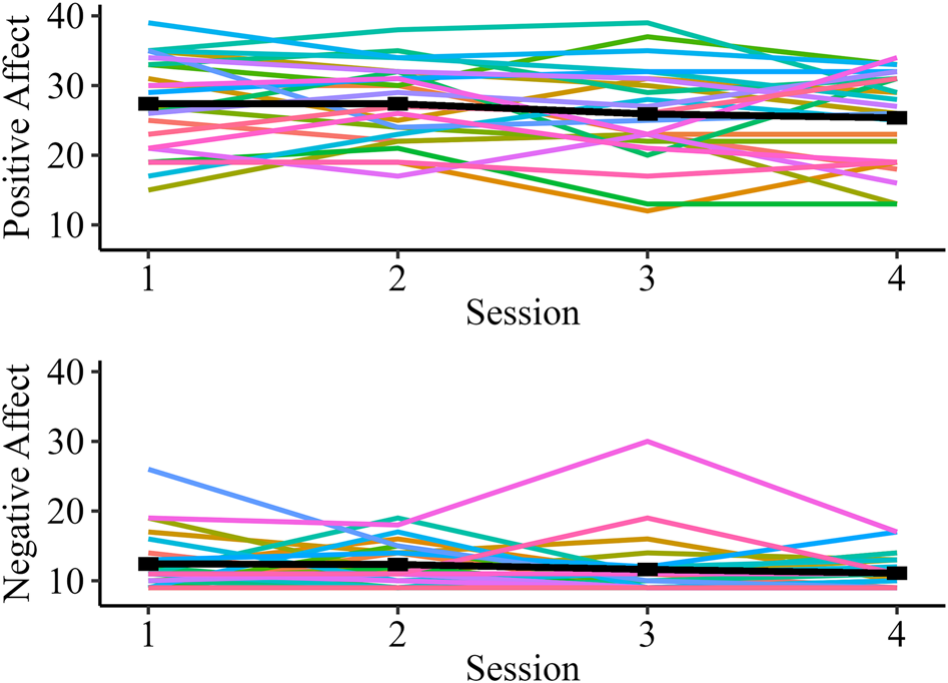
Visualization of positive and negative daily affect across sessions, colored by subject. A higher score indicates greater levels of positive or negative daily affect. Mean scores averaged across subjects at each session are visualized in black.

Summary descriptive statistics for behavioral predictors are summarized in Table 1. Positive daily affect had larger variability across sessions (standard deviation, 6.48) compared with negative daily affect (standard deviation, 3.67), and within-subject variation in positive and negative daily affect (i.e., subject mean centered affect scores) did not associate with each other [*B* (95% CI) = −0.05 (−0.30 to 0.21), *p* *=* 0.71]. Positive and negative daily affect across sessions is visualized in Figure 1.

When considering associations between within-subject daily affect variation and covariates, there were no associations with male sex [positive affect, *B* (95% CI) = −0.37 (−1.68 to 0.95), *p* = 0.58; negative affect, *B* (95% CI) = 0.00 (−1.07 to 1.07), *p* = 1.00], age [positive affect, *B* (95% CI) = −0.01 (−0.20 to 0.18), *p* = 0.92; negative affect, *B* (95% CI) = 0.00 (−0.15 to 0.15), *p* = 1.00], head motion [positive affect, *B* = 2.07 (−22.25 to 26.39), *p* = 0.87; negative affect, *B* (95% CI) = 3.62 (−16.02 to 23.26), *p* = 0.72], or drowsiness [positive affect, *B* (95% CI) = −0.20 (−0.58 to 0.18), *p* = 0.30; negative affect, *B* (95% CI) = 0.13 (−0.18 to 0.43), *p* = 0.41]. Session had a significant negative association with positive daily affect [*B* (95% CI) = −0.75 (−1.32 to −0.18), *p* = 0.01] and negative daily affect [*B* (95% CI) = −0.47 (−0.94 to 0.00), *p* = 0.05]. Participants reported difficulty staying awake for 55/276 observations (*n* *=* 32 low-demand videos, *n* = 10 narrative movies, *n* = 13 non-narrative clips).

### Univariate network effects

**Figure 2.**
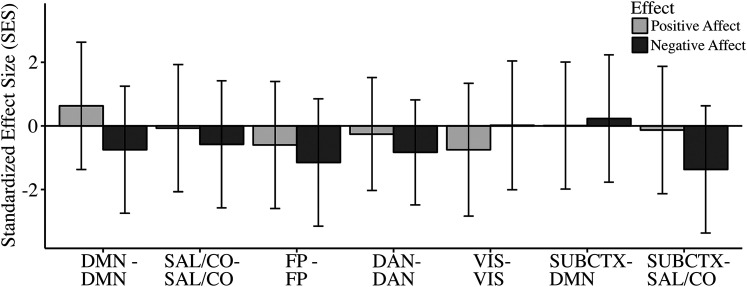
Daily affect in relation to within-network connectivity of hypothesized networks. Associations between positive daily affect (light gray bars) and negative daily affect (dark gray bars) in relation to univariate within-network connectivity of hypothesized networks, with error bars representing 95% confidence intervals. Network-level SES statistics were calculated as the difference between the network-level observed effect and the mean of the network-level permuted null, over the standard deviation of the network-level permuted null. SES, standardized effect size; DMN, default mode network; SAL/CO, salience/cingulo-opercular network; FP, frontoparietal network; DAN, dorsal attention network; VIS, visual network; SUBCTX, amygdala and nucleus accumbens.

When considering positive affect, there were no significant associations between positive daily affect and within-network connectivity of default mode, salience/cingulo-opercular, frontoparietal, dorsal attention, or visual networks. There were additionally no significant associations between positive daily affect and connectivity between subcortical affective nodes (amygdala and nucleus accumbens) and default mode or salience/cingulo-opercular networks. When considering negative affect, there were also no significant associations between negative daily affect and within-network connectivity of default mode, salience/cingulo-opercular, frontoparietal, dorsal attention, or visual networks. There were additionally no significant associations between negative daily affect and connectivity between subcortical affective nodes and default mode or salience/cingulo-opercular networks. Results were unchanged when excluding runs with a high likelihood of sleep. Effect sizes, 95% confidence intervals, and probability values for hypothesized effects are reported in Table 2. The degree of uncertainty in hypothesized effect size estimates is visualized in Figure 2.

**Table 2.** Positive and negative daily affect and hypothesized univariate network connectivity

Network	Positive affect			Negative affect		
	*t* (95% CI)	SES (95% CI)	*p*_one-tailed_	*t* (95% CI)	SES (95% CI)	*p*_one-tailed_
DMN–DMN	0.42 (−0.92 to 1.77)	0.63 (−1.37 to 2.63)	0.26	−0.37 (−1.41 to 0.68)	−0.75 (−2.75 to 1.25)	0.23
SAL/CO–SAL/CO	−0.06 (−1. 67 to 1.55)	−0.07 (−2.07 to 1.93)	0.49	−0.36 (−1.72 to 1.00)	−0.58 (−2.58 to 1.42)	0.27
FP–FP	−0.37 (−1.47 to 0.74)	−0.60 (−2.60 to 1.40)	0.28	−0.73 (−1.95 to 0.50)	−1.15 (−3.15 to 0.85)	0.13
DAN–DAN	−0.30 (−2.00 to 1.40)	−0.26 (−2.03 to 1.52)	0.40	−0.69 (−2.05 to 0.67)	−0.83 (−2.48 to 0.82)	0.21
VIS–VIS	−0.68 (−2.74 to 1.38)	−0.75 (−2.84 to 1.34)	0.22	0.00 (−1.75 to 1.75)	0.02 (−2.01 to 2.04)	0.50
SUBCTX–DMN	−0.02 (−1.26 to 1.21)	0.01 (−1.99 to 2.01)	0.50	0.08 (−0.89 to 1.06)	0.23 (−1.77 to 2.23)	0.42
SUBCTX–SAL/CO	−0.05 (−1.04 to 0.95)	−0.13 (−2.13 to 1.87)	0.46	−0.65 (−1.63 to 0.32)	−1.37 (−3.37 to 0.63)	0.10

Positive and negative daily affect in relation to hypothesized univariate network connectivity patterns. Network-level SES statistics were calculated as the difference between the network-level observed effect and the mean of the network-level permuted null, over the standard deviation of the network-level permuted null. *t*, network-level *t*-statistic calculated by averaging *t*-statistics across network edges; SES, standardized effect size; *p*_one-tailed_, one-tailed uncorrected probability value following 1,000 permutations; DMN, default mode network; SAL/CO, salience/cingulo-opercular network; FP, frontoparietal network; DAN, dorsal attention network; VIS, visual network; SUBCTX, amygdala and nucleus accumbens.

**Figure 3.**
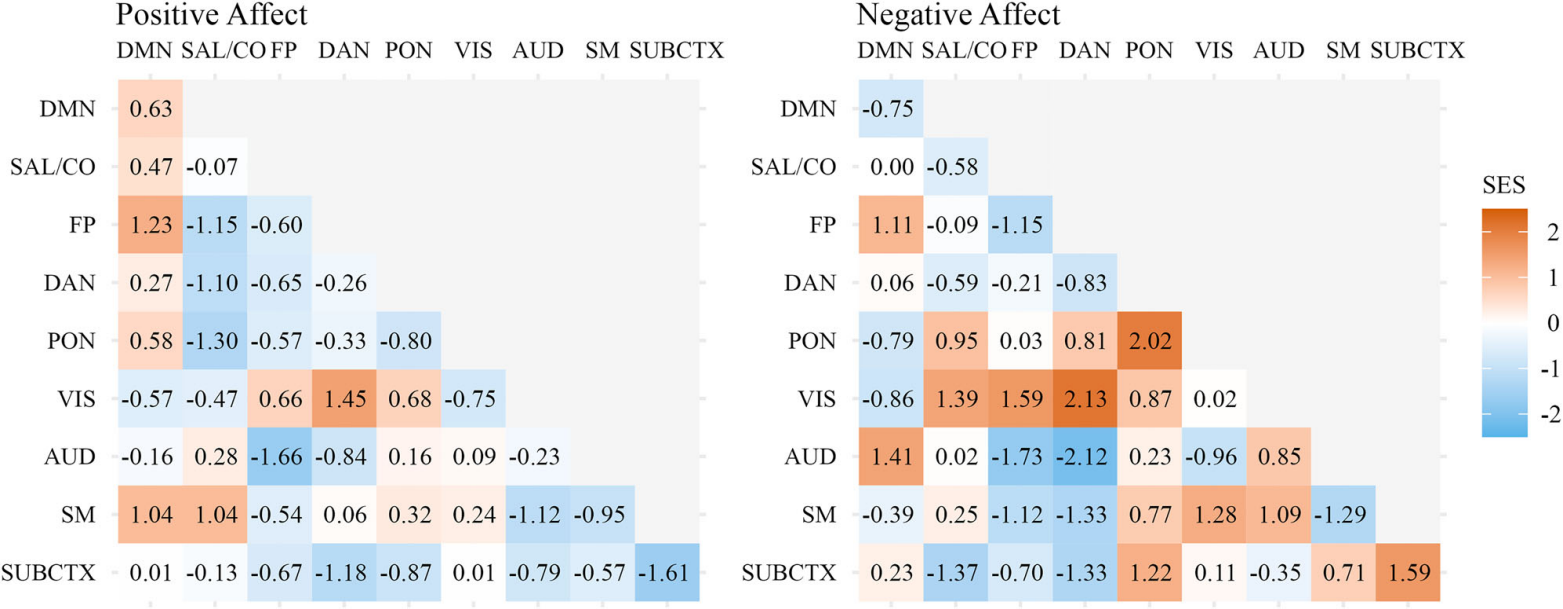
Exploratory effects of daily affect on network-level connectivity. Standardized effect sizes were calculated as the difference between the network-level observed effect and permuted network-level null mean over the permuted network-level null standard deviation. SES, standardized effect size; DMN, default mode network; SAL/CO, salience/cingulo-opercular network; FP, frontoparietal network; DAN, dorsal attention network; PON, parietal occipital network; VIS, visual network; AUD, auditory network; SM, sensorimotor network; SUBCTX, amygdala and nucleus accumbens.

In an exploratory analysis, effects were estimated for all within- and between-network connections. Positive daily affect did not significantly associate with any network-level functional connectivity patterns. Negative daily affect showed three network-level associations at *p* ≤ 0.05 uncorrected, none of which survived FDR correction, for between-network connectivity of dorsal attention network (DAN) to auditory network [*t* (95% CI) = −2.01 (−3.89 to −0.14), SES (95% CI) = −2.12 (−4.12 to −0.12), *p*_two-tailed_ *=* 0.03, *p*_FDR_ = 1.000], dorsal attention network to visual network [*t* (95% CI) = 2.10 (0.11–4.10), SES (95% CI) = 2.13 (0.13–4.13), *p*_two-tailed_ *=* 0.03, *p*_FDR_ = 0.74] and within-network connectivity of parietal occipital network [*t* (95% CI) = 2.41 (0.15–4.66), SES (95% CI) = 2.02 (0.14–3.90), *p*_two-tailed_ = 0.04, *p*_FDR_ = 0.62]. Results were unchanged when excluding runs with a high likelihood for sleep. Effect size estimates across all exploratory patterns of network-level connectivity are visualized in Figure 3.

### Multivariate network effects

Multivariate cNBS (mv-cNBS) did not show a significant relation between positive daily affect and a multivariate network pattern at the whole-brain level (when considering all within- and between-network connections), across all within-network connections, nor across all between-network connections. Across all network groupings, when considering positive daily affect, the null effect was larger than the observed effect, and as such standardized effect sizes could not be calculated. A multivariate effect was also not seen using mv-cNBS for negative daily affect at the whole-brain level (SES = 1.00, *p* = 0.16), across all within-network connections (SES = 1.10, *p* = 0.13), nor across all between-network connections (SES = 0.64, *p* = 0.26). As no effects were significant, uncorrected *p*-values are reported.

### Proportional variance explained

**Figure 4.**
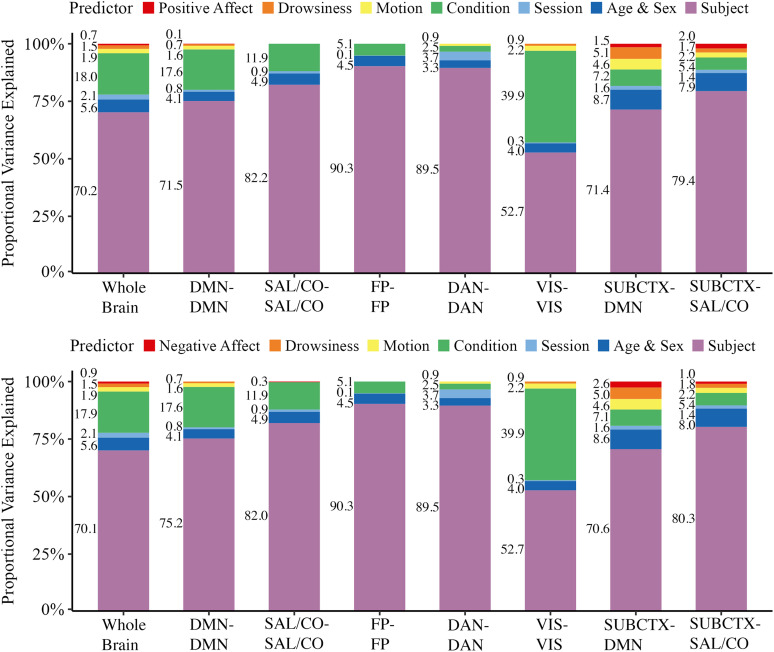
Proportional variance explained by daily affect and common covariates. The contribution of positive and negative daily affect in comparison with other predictor variables for explaining functional connectivity variance, considered at the whole-brain level (all within- and between-network connections) and for hypothesized networks. Positive and negative daily affect were considered in separate mixed-effects models and improvement in model *r^2^c* was assessed following stepwise addition of predictor variables which were then represented proportionally to the total variance explained. Any variables not visualized had a negative impact on model *r^2^c*. DMN, default mode network; SAL/CO, salience/cingulo-opercular network; FP, frontoparietal network; DAN, dorsal attention network; VIS, visual network; SUBCTX, amygdala and nucleus accumbens.

The largest overall contributor to explaining functional connectivity variance was subject identity, which accounted for 52.73–90.30% of the variance across analyses. Demographic variables (age and sex) accounted for an additional 3.33–8.66% of the variance. The viewing condition variable accounted for between 2.53% of the variance in the dorsal attention network and 39.87% of the variance in the visual network. Positive daily affect accounted for 0.67% of the variance at the whole-brain level (i.e., across all edges), 0.14% of the variance within the default mode network, 1.49% of the variance between subcortex and default mode network, 2.02% of the variance between subcortex and salience/cingulo-opercular network, and negatively impacted variance explained for the other hypothesized networks. Negative daily affect accounted for 0.88% of the variance at the whole-brain level, 0.29% of the variance within salience/cingulo-opercular network, 2.58% of the variance between subcortex and default mode network, and 0.96% of the variance between subcortex and salience/cingulo-opercular network, while negatively impacting variance explained for the other hypothesized networks. Session, head motion, and drowsiness confounds each accounted for a small (0.00–5.06%) amount of functional connectivity variance. A summary of proportional variance explained by each predictor is visualized in Figure 4.

## Discussion

In this study, self-reported ratings of daily (past 24 h) positive and negative affect were investigated for associations with functional connectivity in a nonclinical community sample using a repeated, dense sampling design. We report small effect sizes which did not reach significance at the level of individual hypothesized networks and when considering multivariate effects across networks. Finally, we show that daily affect either accounted for no or small (<3%) amounts of variance in functional connectivity compared with other sources of variance including individual identity, which is not accounted for in cross-sectional designs. These findings suggest that daily affect variation has a relatively small impact on functional connectivity in healthy adults. Here, we consider these findings alongside prior work and offer suggestions for future studies applying dense sampling to investigate stable varying states.

Based on a prior functional connectivity study of affect variation in a single individual (Mirchi et al., 2019) and meta-analyses on evoked emotions (Barrett and Bliss-Moreau, 2009; K. A. Lindquist et al., 2012; Barrett and Satpute, 2013), we first considered positive and negative daily affect for hypothesized effects on within-network connectivity of default mode, salience/cingulo-opercular, frontoparietal, dorsal attention, and visual networks alongside connectivity of subcortical affective nodes (amygdala and nucleus accumbens) with default mode and salience/cingulo-opercular networks. We report no association between positive or negative daily affect in relation to any of the hypothesized network-level connectivity patterns considered. In our exploratory analyses, we observed an uncorrected association between negative daily affect and between-network connectivity of dorsal attention to the visual network, dorsal attention to the auditory network, and within-network connectivity of the parietal occipital network. Although these findings did not survive multiple-comparisons correction in the context of many comparisons here, they could be used to derive hypotheses for future work. More generally, our results point to relatively small or negligible effects of normative daily affect variation on univariate network functional connectivity. Affect is a broad and integrative process with widely distributed associations across the brain (for reviews, see Duncan and Barrett, 2007; Barrett and Bliss-Moreau, 2009). We therefore considered several multi-network hypotheses here, reporting no associations between positive and negative daily affect and pooled multivariate network patterns when assessed with mv-cNBS.

These findings contrast with previously reported associations between session-to-session affect and functional connectivity (Mirchi et al., 2019). As the study by Mirchi and colleagues examined a single individual over 73 sessions, it's likely that there was increased sampling variability across diverse affective states. Assessing a single subject with this many sessions would additionally increase the power for modeling within-subject variation, though generalizability in a multi-subject context could not be examined. It could be that previously reported relations between session-to-session affect and functional connectivity were true but small effects that the present study was not sufficiently powered to detect. Alternatively, it may be that the generalizability of this pattern is limited across individuals. When considering generalizability, this previous work used a data-driven method to identify affect-associated functional network patterns (Mirchi et al., 2019) which could be more sensitive to individual-specific associations. Other work has highlighted that distinct inter-individual functional network patterns should be considered to detect associations with other variable states like mind-wandering (Kucyi et al., 2024). Given several limitations of single-subject modeling and *n*-of-1 studies, including generalizability and potential feasibility for all populations of interest, we endeavored to expand findings from one such study (Mirchi et al., 2019) to a densely sampled precision cohort with reduced per-participant burden. The null results presented here, at minimum, suggest that single-subject findings related to affect should not be expected to generalize across differences in context and methodology. Future studies with more samples per individual could have statistical power to fit random subject slopes, which may be important to assess whether associations are shared across the group or vary at the individual level. Further knowledge regarding to what extent affect, and other varying states, have individualized representation will illuminate the feasibility of functional connectivity as a biomarker at the level of individual and diagnostic group.

To support planning future studies relating affect and functional connectivity, we examined the relative contribution of daily affect to explaining network-level functional connectivity variance. Across analyses, subject identity explained the greatest magnitude of variation in functional connectivity while daily affect accounted for either no variance or a small (<3%) proportion of the variance. The finding that connectome variance is strongly influenced by individual connectivity patterns, above task activation or session-to-session variability, concurs with prior work (Gratton et al., 2018). Our findings further align with work in clinical patients with major depressive disorder, where group-level clinical features (diagnostic traits and treatment response) accounted for only a small portion of the variance in functional connectivity while >95% of the variance was explained by common shared underlying architecture and individual differences (van der Wijk et al., 2023). We note that in the present study, stable individual features could not be disentangled from shared group features. As the individual effect here was not quantified in proportion to an additional group effect, estimates of the individual effect are inflated as compared with these previous works (e.g., individual associated variance at ∼35–40% in Gratton et al., 2018). Nevertheless, the results presented here, in addition to reports from previous studies (Gratton et al., 2018; van der Wijk et al., 2023), suggest that daily variations are expected to explain small amounts of functional connectivity variance while stable individual and group effects will have the largest impact on functional connectivity variance.

Participants in the present study rated positive and negative affect by reflecting on the past 24 h, mirroring many measures of affective disorder symptoms which are often reflective as well (Furukawa, 2010; Cerimele et al., 2019). While null results were reported here when affect was considered at the level of daily assessment, that doesn't preclude that state-like changes can be detected in the brain at more granular timescales. This includes functional connectivity changes in response to evoked emotions (Barrett and Bliss-Moreau, 2009; K. A. Lindquist et al., 2012; Barrett and Satpute, 2013) or reports that functional connectivity within a session can be associated with moment-to-moment changes in arousal (Chang et al., 2013a, b). The results presented here, alongside previous work, suggest that future studies aiming to use functional connectivity as a biomarker may wish to consider functional connectivity at either the stable individual or moment-to-moment timescales. However, the clinical relevance of moment-to-moment fluctuations is less clear, as affective disorders are defined by prolonged or pervasive affective states lasting over longer timescales (VandenBos, 2015). If daily variability in cohort designs therefore remains of interest, researchers will likely need to plan appropriately in anticipation of small effect sizes.

The results presented here suggest small or negligible effects for the association between functional connectivity and daily affect variation. These results additionally emphasize previous reports that individual identity has a large influence on functional connectivity (Gordon et al., 2017; Gratton et al., 2018) despite partially accounting for inter-individual variation in functional network borders with a consensus parcellation here (Dworetsky et al., 2021; Hermosillo et al., 2024). Future studies aiming to model associations between functional connectivity and day-to-day variations will likely need to anticipate similarly small effect sizes and power their studies accordingly. This could be done by collecting more samples from fewer individuals to simultaneously increase within-subject variance while decreasing between-subject variance. In the previous study of a densely sampled individual, 73 sessions of data were collected to identify an association between functional connectivity and affect (Mirchi et al., 2019). A recent precision imaging study of depression additionally reported associations between salience network connectivity and within-individual variability in anhedonia, which was detected within two participants with sufficient imaging data for single-subject modeling (62 and 39 sessions per participant; Lynch et al., 2024). In sum, investigations regarding associations between functional connectivity and day-to-day variations will likely need to carefully consider their sampling strategy to include more sessions per participant even if that reduces total sample size and subsequent generalizability.

Strengths of our approach included using several methods to increase power for detecting brain-behavior associations, such as the use of movies over resting-state imaging (Finn and Bandettini, 2021), selection of statistical tools shown to have elevated sensitivity for network-level inference (Noble and Scheinost, 2020; Noble et al., 2022), and efforts to increase precision in network-level functional connectivity estimates with a probabilistic atlas. However, there are a few limitations to be considered. In the community sample recruited for the present study, the variance of both positive and negative daily affect was likely reduced compared to other populations of interest, in particular mental health and affective disorders. Given the relation between effect size, power, and sampling variability (Meyvis and Van Osselaer, 2018), reduced variance in affect here would impact sensitivity to detect an association between affect and functional connectivity. It's unclear whether the present study design would have been more sensitive to a relation between affect and functional connectivity if a clinical population with higher affect variability had been recruited such as patients with bipolar II disorders (Henry et al., 2001) or if we had considered a different feature with greater variability such as suicidal ideation in a clinical sample (Kleiman et al., 2017). Future studies examining within-individual variation in affect and other states should carefully consider behavioral assessment selection and weigh trade-offs between sensitivity to state-like variability and test-retest reliability. Parcellation choice can also greatly impact reported associations between functional connectivity and behavior (Bryce et al., 2021), and it is unclear to what degree divergent findings here could be related to previous studies reporting regional-specific results obtained using finer-grained parcellations (Mirchi et al., 2019).

Dense sampling and precision imaging study designs have been suggested as a candidate method for identifying associations between brain function and time-varying clinical states, such as affect. We applied a dense sampling protocol in a community sample of adults and found that associations between daily affect and functional connectivity across levels of network inference were generally small. In addition, daily affect either accounted for no proportional variance in functional connectivity or accounted for a small degree of the variance (i.e., 3–125× smaller than the viewing condition). Future work aiming to assess links between functional connectivity and variable affect in clinical samples should carefully consider expected effect sizes, sampling strategy, and timescales of measurement to maximize study power.

## Synthesis

Reviewing Editor: Alexander Soutschek, Ludwig-Maximilians-Universitat Munchen Decisions are customarily a result of the Reviewing Editor and the peer reviewers coming together and discussing their recommendations until a consensus is reached. When revisions are invited, a fact-based synthesis statement explaining their decision and outlining what is needed to prepare a revision will be listed below. The following reviewer(s) agreed to reveal their identity: Aaron Kucyi.

In this paper, the authors report an fMRI study of 24 individuals who underwent four neuroimaging sessions as well as self-report measures of positive and negative affect on each day of scanning. Analyzing functional connectivity (FC; estimated within each 6.5-minute fMRI run) between prespecified regions implicated in affective networks and performing more exploratory analyses, they report that day-to-day variations in self-reported affect largely does not explain variance in FC at the group level.

It is important to understand how day-to-day fluctuations in affect are reflected in brain activity and more specifically impact FC estimates, especially when considering that FC is actively being studied and considered as a trait-level biomarker of psychiatric illness. The paper is well-written and reports data analyses that are technically sound. However, I have concerns about how the authors are interpreting the findings and the overall message that is being conveyed (including recommendations for the field). There are also limitations in the study design and sample that should preclude strong conclusions regarding the relationship between affect states and FC.

Major concerns:

- While the data support a lack of relationship between FC and day-to-day variations in PANAS reports, this obviously does not mean that affect variations are not represented in the brain. The lack of relationship may be a product of the time scale at which PANAS ratings and fMRI scans were obtained. Experiences of affect fluctuate across various time scales, including within a single day and even on time scales of hours and minutes or less. As such, the single PANAS rating obtained at each visit may not be granular enough to capture relationships with FC dynamics. Due to these concerns, I believe that conclusions regarding the lack of relationship between FC and within-subject affect variation should be toned down and should always come with clear qualifiers regarding the time scale of measurements throughout the manuscript.

- It appears that several subjects showed minimal variation in PANAS ratings across sessions. The negative affect ratings are especially 'flat' across sessions (Fig. 1) in a large proportion of the sample. Of course relationships between FC and affect will not be found if there are no variations, or no meaningful variations, in affect. This issue should be better acknowledged throughout the manuscript and potentially addressed in analyses. Were the ratings normally distributed? This is important, considering that the authors main analyses relied on linear mixed effects models.

- Related to issues surrounding time scale of measurements, the authors minimally acknowledged extensive research on dynamic/time-varying FC and its potential relevance to affect fluctuations. For example, rigorous studies have shown that on time scales of tens of seconds, FC dynamics are associated with within-subject arousal (Chang et al., 2013a, 2013b), attention (Rosenberg et al., 2020), and mind-wandering (Kucyi et al., 2021). Moreover, spontaneous variations in affect and emotion during resting states relate to transient fMRI multivariate patterns of activity (Tusche et al., 2014; Kragel et al., 2016). I recommend incorporating these studies in a discussion of relevant time scales at which relationships between FC and affect may be found (see (Lurie et al., 2020) for detailed overview).

- Even if the affect variations reported by subjects are meaningful, it is possible that relationships with FC were not found at the group level due to individual differences in neural representations of these variations (a problem that this study is not powered to addressed). Indeed, recent work shows that FC variations that predict mind-wandering are explained by distinct functional-anatomical patterns in each individual (Kucyi et al., 2024). This issue deserves further attention, especially given points noted above regarding low within-subject variation in affect ratings.

- Overall, the paper and its interpretations are fMRI-centric, as the focus is on FC as a potential biomarker. However, variations in mood and affect at similar time scales (e.g. day-to-day or across hours) have been strongly linked to brain network functional dynamics in human intracranial electrophysiological recordings (Kirkby et al., 2018; Scangos et al., 2021). As such, brain representations of subjective affect variation can be detected at somewhat similar time scales to those reported here. How do the authors reconcile those non-fMRI findings with their results?

Minor concerns

- The introduction defines emotions as "affective experiences related to external stimuli." It should be acknowledged that this is not a universally agreed upon definition, or perhaps a more conventional definition should be stated.

- To clarify, did the authors not perform spatial smoothing?

References

Chang C, Liu Z, Chen MC, Liu X, Duyn JH (2013a) EEG correlates of time-varying BOLD functional connectivity. Neuroimage 72:227-236.

Chang C, Metzger CD, Glover GH, Duyn JH, Heinze H-J, Walter M (2013b) Association between heart rate variability and fluctuations in resting-state functional connectivity. Neuroimage 68:93-104.

Kirkby LA, Luongo FJ, Lee MB, Nahum M, Van Vleet TM, Rao VR, Dawes HE, Chang EF, Sohal VS (2018) An Amygdala-Hippocampus Subnetwork that Encodes Variation in Human Mood. Cell 175:1688-1700.e14.

Kragel PA, Knodt AR, Hariri AR, LaBar KS (2016) Decoding Spontaneous Emotional States in the Human Brain. PLoS Biol 14:e2000106.

Kucyi A, Anderson N, Bounyarith T, Braun D, Shareef-Trudeau L, Treves I, Braga RM, Hsieh P-J, Hung S-M (2024) Individual variability in neural representations of mind-wandering. Network Neuroscience:1-66.

Kucyi A, Esterman M, Capella J, Green A, Uchida M, Biederman J, Gabrieli JDE, Valera EM, Whitfield-Gabrieli S (2021) Prediction of stimulus-independent and task-unrelated thought from functional brain networks. Nature Communications 12:1793.

Lurie DJ et al. (2020) Questions and controversies in the study of time-varying functional connectivity in resting fMRI. Netw Neurosci 4:30-69.

Rosenberg MD, Scheinost D, Greene AS, Avery EW, Kwon YH, Finn ES, Ramani R, Qiu M, Constable RT, Chun MM (2020) Functional connectivity predicts changes in attention observed across minutes, days, and months. Proc Natl Acad Sci U S A 117:3797-3807.

Scangos KW, Khambhati AN, Daly PM, Makhoul GS, Sugrue LP, Zamanian H, Liu TX, Rao VR, Sellers KK, Dawes HE, Starr PA, Krystal AD, Chang EF (2021) Closed-loop neuromodulation in an individual with treatment-resistant depression. Nat Med.

Tusche A, Smallwood J, Bernhardt BC, Singer T (2014) Classifying the wandering mind: revealing the affective content of thoughts during task-free rest periods. Neuroimage 97:107-116.
